# Psychological impact after treatment in patients with head and neck cancer

**DOI:** 10.4317/medoral.25878

**Published:** 2023-02-18

**Authors:** Isabel María Reyes, María José Arenilla, David Alarcón, José Carlos Jaenes, Manuel Trujillo

**Affiliations:** 1 Faculty of Social Sciences, University Pablo de Olavide, Spain; 2 Department of Otolaryngology - Head and Neck Surgery, Virgen de Valme University Hospital, Seville, Spain; 3 School of Medicine, New York University, New York, NY, United States

## Abstract

**Background:**

Cancer is the second cause of death all over the world and it causes considerable morbidity, disability, and treatment sequela, which often lead to post-treatment pain and disFigurement. This study aims to evaluate such physical sequelae, and their psychological, (cognitive and emotional), impact, in a cohort of patients treated for Head and Neck (HNC) cancer, in search for methods to help such patients deal effectively with the psychological effects of their cancer treatments adverse consequences.

**Material and Methods:**

The sample consists of 56 subjects, 47 men and 9 women, ranging from 47 years to 86 years of age, who were treated for head and neck cancers at Spanish Public General Hospital in the Otolaryngology Unit, Surgery Section. Two types of questionnaires were used in the study: the Questionnaire of Sequelae after Treatment of head and neck carcinoma and the State-Trait Anxiety Inventory (STAI-E and R).

**Results:**

With respect to anxiety, the study found high levels of state anxiety which was significantly associated with the degree of perception of social stigma but was not associated with the post-treatment sequelae themselves nor with the level of discomfort that such symptomatic sequelae produced. The presence of a post-surgical stoma with cannula, increased patient’s stigma (both components: external rejection and self-rejection) and state anxiety ratings, while there was no difference in state anxiety between cannulated and non-cannulated patients. There are few differences between men and women in terms of the presence of anxiety and their responses are similar in terms of the after-effects of surgery.

**Conclusions:**

Our study confirmed that current treatments for Head and Neck carcinoma generate adverse symptomatic sequela that impose significant psychological and physical burden for these patients. We will discuss the various pathways for preventive intervention that these findings open up.

** Key words:**Psychology, dysmorphia, surgery, tracheotomy, anxiety.

## Introduction

Cancer all is one of the leading causes of death worldwide. WHO reports that in 2017, 10.08 million, out of the 56 millions fatalities were caused by cancer the second largest cause of mortality after cardiovascular disease. According to the Spanish Register Cancer Net (Red Española de Registro de Cáncer) 280,100 cancers were diagnosed in 2022, 7,779 cases of being Head and Neck cancer (HNC). The significant life disruption experienced by people with HNC is comparable to the one experienced by people suffering other serious chronic illnesses (1). Thus, an initial diagnosis of cancer can be conceptualized as the onset of chronic illness; a biological and biographical disruption in which a person’s assumptions about their current and future life trajectory and sense of self are challenged (2).

According to Stark & House (3), anxiety is common in cancer patient populations, and needs to be recognized early, and managed to a substantial degree by cancer care professionals. Those who are not mental health specialists need to understand the nature of this anxiety, be able to discriminate morbid from normal anxiety and to detect different expressions of anxiety, in order to develop communication strategies which, facilitate the alleviation of such anxiety. Head and neck cancer (HNC) is frequently described as one of the most emotionally traumatic of all cancers. because the facial disFigurement and dysfunction associated with its treatment (4). The prevalence of psychological comorbidities is significantly higher in head and neck carcinoma patients as compared to other ENT patients. Lydiatt *et al*. (5) report that patients with HNC were at an increased risk for developing depression and anxiety, with an incidence of 15–50%, as compared to patients with other forms of cancer.

HNC patients may also suffer variable degrees of functional impairment related to speaking, swallowing, breathing, taste, and smell during treatment. Often the illness course also makes social contact difficult (6). In fact, apart from its impact on physical activities, tracheostomy, -and the insertion of a cannula-, involves prominent disFigurement of the anterior neck altering the patient’s body image perception (BIP) (7). Facial disFigurement has long been viewed as one of the potentially most distressing aspects of head and neck cancer treatment, because of the high visibility and the vital importance of the facial region’s to the development of a positive self-concept, to interpersonal relationships, and to communication in general (8). Surgery often leads these patients to develop unique physiological and psychosocial needs, highlighting the fact that additional research is critical in order to enhance long-term functional recovery after surgical treatment (9). Additionally, HNC patients are vulnerable to psychiatric comorbidities, (particularly anxiety, depression), they worry about health, job and economic problems, experience family tensions, and also suffer often from cancer shame and stigma (10).

Mutilating injuries to the head and neck, more so than to other body regions, are considered triggers for reactive psychological disorders due to the profound changes of body appearance and image that surgery sometimes entail; changes which can produce a cascade of effects in areas such as self-image, relationship with partner(s), social and sexual function, fostering potential social isolation. Permanent stoma in laryngeal cancer patients, by itself, is reported to have a major influence on quality of life (QOL) (7). Studies emphasize the importance of managing physical and psychological symptoms (11), including potential conflicts between patients and family members or intimate partners. Other researchers have emphasized the importance of providing patients psychological information, though many such patients avoid written information or information provided by other than their surgeons,and strongly prefer to obtain it face-to face in direct interaction with them (12).

Anxiety, quality of life and satisfaction with treatment, have been studied by Shiraz, Rahtz, Bhui, Hutchison & Korszun (13) who found that patients with high scores on anxiety and depression reported poorer quality of life, and 40% of those with high levels of psychological distress were willing to consider obtaining psychological support. Though patients report high levels of satisfaction with their medical and surgical care, there were a few exceptions: 1) many have psychological problems and emotional needs which are not being met; 2) that satisfaction with personal appearance remained negative, even after micro-reconstructive surgery had been conducted; 3) certain participants reported changing their jobs because of HNC treatment as a direct adverse consequence of cancer.

The nature of the anxiety experienced by patients through the treatment process is also important, as it determines possible preventive and treatment strategies. In particular, the difference between State vs. Trait anxiety is key. State Anxiety can be defined as a transitory emotional state consisting of feelings of apprehension, nervousness, (emotional component), physiological signs such as an increased heart rate or respiration (physical component) and concerns and worry about a specific situation (cognitive component)(14). State Anxiety reflects the “right” now reaction to a situation. Trait Anxiety refers to the intrinsic and sTable tendency of people to attend to, experience, and report negative emotions such as fear, worries, and concerns across many situations. Trait is a personality characteristic, part of the personality dimension of neuroticism versus emotional stability. It may be based on past experiences but it has a substantial genetic component (15).

## Material and Methods

- Study design

A correlational descriptive design was used. The study was conducted in a Spanish Public General Hospital in the Otolaryngology Unit, Surgery Section. Approval was obtained by the Unit Director. Eligible patients visited the principal investigator and were informed of the purpose of the study, nature of participation and the confidentiality of personal information. All participant subjects were informed of their freedom to withdraw at any time with no consequences for their ongoing treatment. Written informed consent was obtained from all prospective subjects prior to entry into the study. Throughout the course of the investigation, the rights and dignity of all respondents were carefully protected, personal information was protected under a code, and the confidentiality of the people evaluated was guaranteed.

A convenience sample of 56 participants was registered in this study. All participating subjects were head and neck cancer patients (HNC). Due to patient’s age, and socio-cultural conditions, the study informational procedure was conducted in a face-to-face session at the surgery office in order to assure all patients understood questions. When required, physician answered questions and provided a clear description of the purpose of the questionnaire (16). Technical information about their cancer: type of tumor, and surgical procedure proposed was delivered directly by the surgeon. Study tests completion took between 30-45 minutes.

- Instruments

A questionnaire specially designed for the study (“Questionnaire of sequelae after treatment of head and neck cancer”) was used assess cancer patients. The questionnaire included an introduction with the aims of the research, specific instructions to fill it out, the aims of the survey, and required ethics information. In addition to sociodemographic, the questionnaire requested information about location tumor (oral cavity, glottis, hypopharynx, nasopharynx, oropharynx, subglottis, supraglottis, or transglottis); type of treatment (surgery, surgery plus radiotherapy, surgery plus radiotherapy and chemotherapy, radiotherapy, or radiotherapy plus chemotherapy) and timing of the survey from completion of treatment (< 6 months, between 6 months and 1 year, between 1 and 2 years, between 2 and 3 years, between 3 and 5 years, or > 5 years); and if they carried a tracheotomy (cannula).

A group of four questions were included to assess self-rejection like “Do you think that you are no longer the same person as before the intervention?” and five questions were used to evaluate feeling rejected by others like “Do you think/feel that you may be rejected because of your physical changes?”. The patients were required to answer in a Likert scale of 0 (Not at all), 1 (A little), 2 (Quite a bit) and 3 (A lot). Internal consistency for each subscale was measured by Cronbach’s alpha. The reliability of the scale was high for self-rejection (α = .780) and feeling rejected by others (α = .818).

Patients also rated twenty-one questions about level of discomfort with the symptoms like “Double chin increase” or “Change in skin color neck”, with a Likert scale of 0 (Not at all), 1 (A little), 2 (Quite a bit), and 3 (A lot). The reliability of the scale was high (α = .897).

The State-Trait Anxiety Questionnaire (STAI-E and R) was used to measure anxiety. We used a Spanish adaptation by Buela Casal & Guillén-Riquelme (14). The test evaluates separately trait and state anxiety, and is also a very common instrument to evaluate anxiety in health psychology, even in HNC (9). The Spanish version includes forty items; twenty for each subscale and subjects have to answer in a scale of 0 (Almost ever), 1 (Sometimes), 2 (Often) and 3 (Almost always). Trait anxiety items are of the type of “I am a calm person or I would like to be as happy as the others”; while those of state anxiety are of the type of “I feel nervous or I feel anxious”. Spanish version reliability is very high: internal consistency 0.90-0.93 for State subscale, and 0.84-0.87 for Trait subscale. Correlations with other anxiety assessment questionnaires are also considerer high, Taylor Manifest Anxiety and Cattel Anxiety Scale (15) (0.73-0.85). In this study, the reliability of the scale was high for STAI-State (α =.955) and STAI-Trait (α =.893).

- Statistical analysis

Statistical analysis was conducted Jamovi Version 2.2 software. The categorical variables were described by frequencies and percentages, and the quantitative variables were described by mean and standard deviation. Welch’s t-test were used to compare the means of not-cannula versus cannula patients on quantitative variables. Violin Plots were used to compare state anxiety, trait anxiety and total anxiety score in patients with cannula and without cannula. Pearson’s correlation coefficient was used to assess how strong the relationship was between quantitative variables (age, total rejection, self-rejection, rejected by others, discomfort with symptoms, state anxiety, trait anxiety and total anxiety). Also, a linear regression model was used to analyze the factors predicting patients' state anxiety. The number of respondents was determined according to the G * Power program (17). At least 56 subjects would be required to apply linear regression in the analysis of up to 5 predictor variables for α = 0.05 and power 1-β = 0.95. Since all of the participants did not answer all the questions, the sample size varied for different results (ADLs). A value of *p* < 0.05 is considered statistically significant.

## Results

- Descriptive

The sample consisted of 56 participants with age range from 47 years to 86 years. 47 of the participants were men (83.93%) with a mean age of 66.5 [10.2] years, and 9 were women (16.2%) a mean age of 60.9 [6.37] years. Of all participants, 20 men (35.7%) and 2 women (3.6%) carried a cannula. Frequencies and percentages of categorical variables about characteristics of the patients are given in Supplement 1. Supplement 2 shows the symptoms together with the level of discomfort of the patients with them.

- Not cannula versus cannula patients

The Welch’s t-test (Table 1) demonstrated statistically significant differences between not cannula versus cannula patients on total rejection (tWelch = -3.569, *p* < .01), self-rejection (tWelch = -2.777, *p* < .01), rejected by others (tWelch = -3.189, *p* < .01), discomfort with the symptoms (tWelch = -2.410, *p* < .05) and on state anxiety (tWelch = -2.819, *p* < .01). No statistically significant differences between not cannula versus cannula patients are found on trait anxiety (tWelch = -0.908, *p* = .369), total anxiety (tWelch = -1.962, *p* = .057) or age (tWelch = 0.639, *p* = .526).

- Pearson’s correlation coefficients of quantitative variables

Pearson’s correlation coefficients showed strong and significant relationships between all variables analyzed (total rejection, self-rejection, rejected by others, discomfort symptoms, state anxiety, trait anxiety and total anxiety) with the exception of the age variable, which was not related to any of the variables analyzed (Table 2).

- Regression analysis of State Anxiety predictors

Linear regression model showed that the factors predicting patients' state anxiety accounting for 84% of explained variance (Table 3). In the model, age and sex of participants did not predict a significant effect on state anxiety (ps > .05). Trait anxiety significantly accounted for the variance of state anxiety (β = .404, *p* < .001). Controlling for all other factors, the level of perceived total rejection (self and others) (β = .304, *p* < .001) and the level of discomfort with symptoms (β = .314, *p* < .01) significantly predicted state anxiety.

**Table 1:**
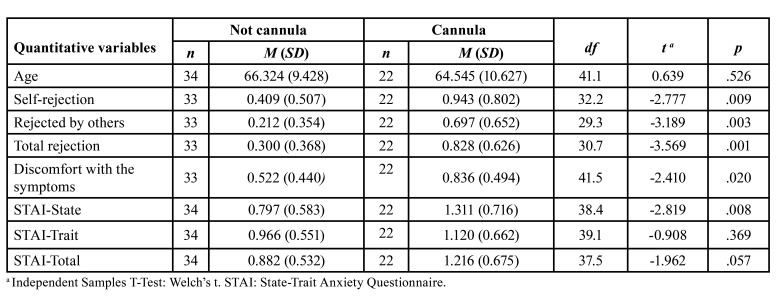
Differences between not cannula versus cannula patients on quantitative variables.

**Table 2:**
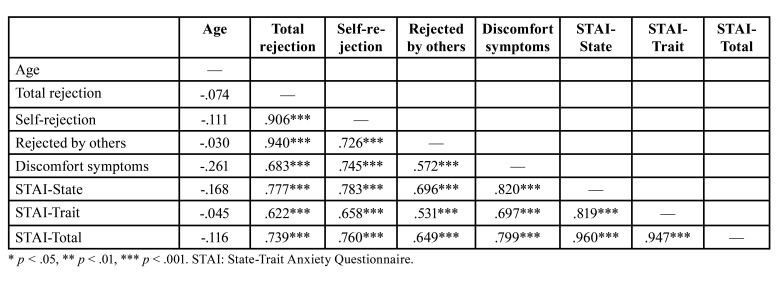
Correlation matrix of quantitative variables.

**Table 3:**
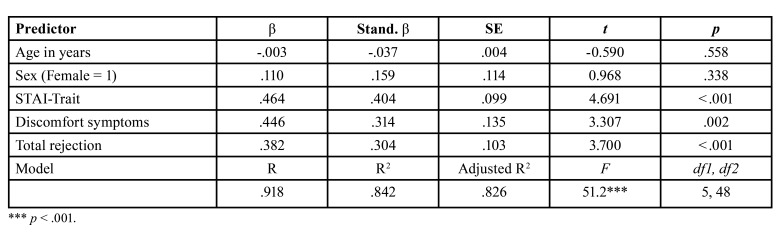
Regression analysis for variables predicting State Anxiety.

## Discussion

In line with the findings of the research literature (3,4,6), our study confirms the high physical and emotional morbidity that survivors of HNC suffer. Stigma, both self and other generated, is high and predicts high levels of trait anxiety as well as a cascade of functional effects in other areas of the patients’ lives.

Living with cannula has demonstrated to be an important stigma for patients, we found statistically significant differences between not cannula versus cannula patients. Those who live with cannula feel significantly higher levels of self-rejection, rejection by others, discomfort with the symptoms and higher levels of state anxiety, in line with the findings of Gilony *et al*. (7). This study also demonstrated a significant reduction in life satisfaction among cannulated patients. Of interest, there is no difference in Trait anxiety between Cannulated and non-cannulated patients. All the anxiety variance in Anxiety between these groups is due to State Anxiety and thus presumably responsive to the many effective techniques in the armamentarium developed by psychiatry and psychology, over the last few decades, for the treatment of anxiety.

Dropkin (9) suggest that anxiety may continue to escalate in a linear fashion long after treatment has concluded. Thus, the possible effect of postoperative anxiety on self-care and resocialization behavior during the early period after surgery as, well as its development over time, require further examination. The state of the art of the treatment of Anxiety today supports a confidently assertive clinical decision to evaluate the trait anxiety of all surgically treated HNC patients and the provision of Anxiety treatment to those patients who are at risk for disabling high levels of anxiety and/or of anxiety chronification.

According to Buela-Casal and Guillén-Riquelme (14), trait anxiety has been defined as an individual's predisposition to respond with anxiety to challenging adaptational situations, and state anxiety as a transitory emotion characterized by physiological arousal and consciously perceived feelings of apprehension, dread, and tension. It can be said that the distinction between trait and state anxiety is analogous to the distinction between potential and kinetic energy.

A study (18) at the Kings College in London, taking a behavioral genetics view, generally supports the view that state and trait anxiety represent environmentally and genetically mediated aspects of anxiety. Furthermore, the results of the study are consistent with the idea that this relationship is expressed through trait anxiety which is then manifested as state anxiety under threatening circumstances. A likely candidate for genetic influence on anxiety includes a functional polymorphism located in the promoter region of the serotonin transporter gene, which has been linked with phenotypic measures of neuroticism, harm avoidance and anxiety (19). Recent studies suggest that this gene also moderates the effects of negative life events on depression symptoms in adults and adolescents (20). Liu (21) asserts that the manner in which this this gene is expressed through different neurobiological and psychological processes to influence behavioral endophenotypes such as state and trait anxiety will require additional collaborative multidisciplinary research.

In our study, we also found significant levels of elevated trait anxiety, and discovered that trait anxiety is a mayor predictor of state anxiety. This finding may be sufficient to cause an routine practice in the holistic care of HN patients: the routine screening for trait anxiety of these patients, and, if a morbidity threshold is met, to approach them with preventive strategies or actual treatment protocols, so as to keep in optimal check the expected additional development of state anxiety in the post-surgical period and of its chronification.

Patient Satisfaction

In the last few decades, patient satisfaction, a subjective judgement of a person on his or her own course of life, has become an important end point in healthcare quality assessment, as both, satisfaction or dissatisfaction with care can influence patient behavior and impact on the outcome of care. Patient satisfaction can be defined as a positive affective response from the respondents who deem that their care is fulfilling expectations, needs or desires. It must be stressed that these expectations are subject to many changes during the course of the cancer treatment journey (16). Shiraz, Rahtz, Bhui, Hutchison & Korszun (13) a found that, though like in our study, patients report high levels of satisfaction with their medical and surgical care, many live with unmodified psychological problems and with other unmet needs. Henry at al. (22,23) suggested that, in line with hospital resource allocation and cost-effectiveness philosophies, clinicians may contemplate responding to some of such unmet needs by screening patients for high levels of anxiety, specially trait anxiety; appropriate pain management, and using adequate psychoeducation about expected vs possible treatment results in the aftermath of surgery. Support regarding anxiety, changes in sexual functioning, and fear of death and dying (23) would complement a very comprehensive and holistic lan of care.

Stigma and other co-morbidities

In patients holding longer-term survivorship, stigma is significantly correlated with anxiety and depression in HNC patients (12). Proper identification of comorbidities and addressing directly the experience of stigma, should be included in mental health efforts among for patients suffering from HNC.

Combining quantitative clinical assessments and qualitative data, the present study provided a comprehensive understanding of and explanations for, HNC patients’ discomfort with symptoms, stigma, and anxiety. The clinical findings suggested that (1) discomfort with symptoms was significantly associated with stigmatization; (2) more stigmatization correlated with more severe anxiety; identification and the reduction of stigma should be considered for inclusion in evolving protocol of mental health efforts on behalf of HNC patients (10). A number of protocols deserve experimental testing: stigma focused CBT, and skill promotion interventions have demonstrated efficacy in similar clinical situations (11). An educational intervention based on building self-management skills, improved quality of life and reduced anxiety and depression in the experimental group compared with a control group in Brazilian study (24).
